# Completed clinical trials of p53-targeted therapeutic strategies in cancer: A cross-sectional ClinicalTrials.gov registry analysis

**DOI:** 10.1097/MD.0000000000050949

**Published:** 2026-10-16

**Authors:** Nasser M. Alorfi, Nouf M. Alourfi

**Affiliations:** a Pharmacology and Toxicology Department, College of Pharmacy, Umm Al-Qura University, Makkah, Saudi Arabia; b Department of Chemistry, Arts and Sciences College, Rabigh Campus, King Abdulaziz University, Jeddah, Rabigh, Saudi Arabia.

**Keywords:** adenoviral vector, cancer, gene therapy, p53-targeted therapeutic strategies, registry analysis, targeted therapy, TP53

## Abstract

The tumor suppressor gene TP53 is one of the most frequently altered genes in human cancers and plays a central role in regulating cell cycle arrest, apoptosis, and genomic stability. Therapeutic strategies targeting the p53 pathway have been investigated to restore or exploit p53 function in human malignancies. However, the characteristics, reporting completeness, and clinical development patterns of completed trials evaluating p53-targeted therapeutic strategies have not been comprehensively summarized. This study aims to characterize the design, therapeutic strategies, reporting completeness, and primary objectives of completed clinical trials investigating p53-targeted therapeutic strategies for cancer. A cross-sectional registry analysis of ClinicalTrials.gov was conducted using the search terms “Cancer” AND “p53 gene therapy.” Completed studies available as of 1 July 2025 were screened for eligibility. Trials investigating p53-targeted therapeutic strategies, including p53 gene therapy and other therapeutic approaches directly targeting the p53 pathway, were included. Data extracted included study design, cancer type, therapeutic strategy, clinical phase, enrollment, and primary outcome measures. Descriptive statistics were used to summarize study characteristics; no inferential statistical analyses were performed. Twenty-three completed studies met the eligibility criteria, including 20 (87.0%) interventional and 3 (13.0%) observational studies. The included studies evaluated diverse p53-targeted therapeutic strategies, including adenoviral vector-mediated p53 gene therapy, chemotherapy combinations, immunotherapeutic approaches, biomarker-guided treatment strategies, and molecularly targeted therapies. Early-phase clinical development predominated, with Phase I studies accounting for the largest proportion of completed trials. Enrollment information was available for 17 (73.9%) studies, whereas six (26.1%) did not report participant numbers. Primary outcome measures were reported for 15 (65.2%) studies, while eight (34.8%) lacked clearly defined primary outcomes. Five studies (21.7%) had publicly posted study results on ClinicalTrials.gov, whereas 18 (78.3%) had no posted results available. Reported primary endpoints predominantly focused on safety, dose escalation, treatment feasibility, and biomarker evaluation. Completed clinical trials investigating p53-targeted therapeutic strategies remain predominantly in the early stages of clinical development and have primarily focused on evaluating safety, feasibility, and preliminary biological activity rather than definitive treatment efficacy. Variability in registry reporting, including incomplete enrollment, primary outcome, and study result reporting, limits comprehensive assessment of the current evidence base.

## 1. Introduction

Cancer is a leading cause of morbidity and mortality worldwide, driven by complex genetic mechanisms that contribute to tumor initiation, progression, and the development of various aggressive human malignancies.^[1,2]^ p53, encoded by the TP53 gene, is a central transcription factor regulating cell cycle arrest, apoptosis, and DNA repair in response to cellular stress.^[3–5]^ Among the most frequently mutated genes in human cancers is the tumor suppressor gene TP53.^[6,7]^ Mutations in TP53 result in dysfunctional proteins that are unable to perform these tumor-suppressive functions, contributing to the development of a wide range of cancers including breast, lung, bladder, and hematologic malignancies.^[4,8]^ Recent advancements in cancer therapy have increasingly focused on restoring or exploiting the function of p53, a critical tumor suppressor frequently mutated in cancers.

Several strategies are being explored to exploit the p53 pathway. These include MDM2 and MDMX inhibitors, which prevent the degradation of WT p53 in cancers where TP53 is not mutated but functionally suppressed.^[9–11]^ Furthermore, efforts to target mutant forms of p53are ongoing, although challenging due to their heterogeneity. Synthetic lethality approaches offer another avenue, selectively targeting vulnerabilities in TP53-mutated cells.^[12,13]^ Moreover, mutant p53-based adoptive cell transfer (ACT), a form of cancer immunotherapy, has shown promise in treating advanced malignancies by directing immune cells to recognize and eliminate tumor cells expressing mutant p53.^[14,15]^ However, these therapies often face resistance from the tumor microenvironment (TME), particularly in solid tumors,^[16,17]^ highlighting the need to address immunosuppressive barriers for successful clinical translation.

One promising approach in cancer treatment is gene therapy, specifically targeting the restoration of p53 function. Since many tumors harbor defective or mutated TP53, therapeutic strategies have emerged to reactivate p53 and restore its tumor-suppressive activities.^[18,19]^ The central role of TP53 in cancer biology makes it an attractive target for pharmacological intervention, with ongoing efforts aimed at restoring wild-type p53 function or selectively targeting mutant p53 to inhibit tumor growth.^[20–22]^ A variety of vectors, particularly adenoviral vectors such as Ad5CMV-p53, have been explored to deliver the functional gene into tumor tissues. Initial clinical trials conducted over the past two decades aimed to determine the safety, tolerability, and preliminary efficacy of this approach, often in combination with conventional treatments such as chemotherapy, radiation, or surgery.^[23–25]^

Despite the growing body of research on p53 biology and gene therapy, there has been limited comprehensive characterization of completed clinical trials investigating p53-targeted therapeutic strategies in cancer. Understanding the design, reporting characteristics, intervention strategies, and primary objectives of these studies is important for identifying current research trends and gaps in clinical development. Therefore, this cross-sectional registry analysis aimed to systematically characterize completed clinical trials registered on ClinicalTrials.gov that investigated p53-targeted therapeutic strategies for cancer. Specifically, the study describes trial characteristics, including study design, clinical phase, enrollment, cancer types, intervention strategies, reporting completeness, and primary outcome measures, to provide an overview of the current landscape of p53-targeted therapeutic strategies research and inform future clinical investigation.

## 2. Methods

### 2.1. Study design and data source

This cross-sectional registry-based study analyzed completed clinical trials investigating p53-targeted therapeutic strategies for cancer using data obtained from the ClinicalTrials.gov registry. ClinicalTrials.gov is the largest publicly accessible clinical trial registry maintained by the U.S. National Library of Medicine and provides standardized information on study design, participant characteristics, interventions, outcome measures, and study status.

### 2.2. Search strategy

A structured search of ClinicalTrials.gov was conducted using the search terms “Cancer” AND “p53 gene therapy.” The search was performed on 1 July 2025 with the study status filter set to Completed. Both interventional and observational studies were initially screened for eligibility.

### 2.3. Eligibility criteria

Studies were eligible for inclusion if they – were registered as completed on ClinicalTrials.gov; investigated p53-targeted therapeutic strategies for cancer, including adenoviral vector-mediated p53 gene therapy, TP53-guided therapeutic interventions, biomarker-directed therapeutic strategies, immunotherapeutic approaches, molecularly targeted therapies, or combination treatments targeting the p53 pathway; and provided sufficient registry information regarding study characteristics. Duplicate registrations and studies unrelated to p53-targeted therapeutic strategies were excluded. A flow diagram of ClinicalTrials.gov record identification, eligibility assessment, and inclusion is shown in Figure 1.

**Figure 1.**
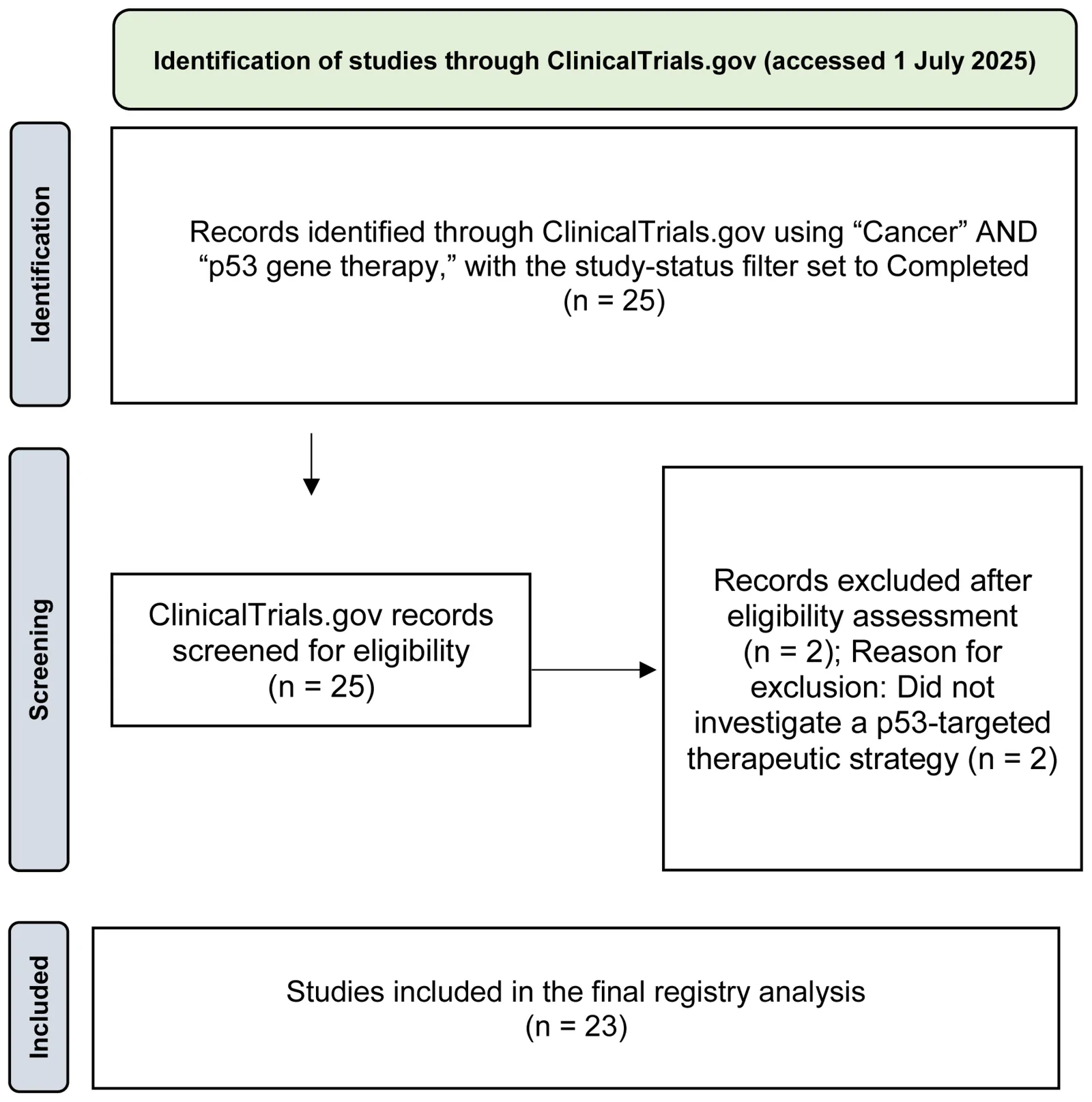
Flow diagram of ClinicalTrials.gov record identification, eligibility assessment, and inclusion.

### 2.4. Study selection and data extraction

Titles, registry records, study summaries, therapeutic strategy/intervention, and outcome descriptions were independently reviewed to confirm eligibility. For each included study, the following information was extracted: National Clinical Trial (NCT) number, study title, cancer type, study design, intervention, clinical phase, enrollment, primary outcome measures, study results availability, and other relevant trial characteristics. Data were extracted directly from ClinicalTrials.gov and compiled in Microsoft Excel for analysis. Data extracted from each eligible study are provided in the Supplementary File S1, Supplemental Digital Content 1.

### 2.5. Missing data

Studies with incomplete reporting of enrollment, primary outcome measures, or other registry variables were retained in the analysis because missing information reflects the current completeness of publicly available trial records. Missing data were reported descriptively and were not imputed or excluded from the analysis.

### 2.6. Statistical analysis

Descriptive statistical analyses were performed using Microsoft Excel (Microsoft Corporation, Redmond, WA, USA). Categorical variables were summarized as frequencies and percentages, while continuous variables were summarized using totals or ranges where appropriate. Missing data were reported separately. No inferential statistical analyses were performed because the objective of this study was to provide a descriptive overview of completed registered clinical trials rather than to evaluate treatment effectiveness or compare study outcomes.

### 2.7. Quality assessment

A formal risk-of-bias or methodological quality assessment was not performed because the objective of this registry-based study was to characterize registered clinical trials and their reporting characteristics rather than to evaluate the efficacy or quality of individual clinical studies.

### 2.8. Ethical considerations

This study analyzed publicly available data obtained from ClinicalTrials.gov and did not involve human participants, identifiable personal information, or access to confidential patient data. Therefore, institutional review board approval and informed consent were not required.

## 3. Results

### 3.1. Study selection and overall characteristics

A total of 23 completed clinical trials investigating p53-related therapeutic strategies in cancer met the eligibility criteria and were included in this registry-based analysis. Of these, 20 (87.0%) were interventional studies and 3 (13.0%) were observational studies. The included studies evaluated a broad spectrum of malignancies, predominantly solid tumors, including lung, breast, ovarian, bladder, colorectal, brain, and oral cavity cancers, in addition to selected hematological malignancies such as acute myeloid leukemia and chronic lymphocytic leukemia.

The clinical characteristics of the included studies are summarized in Table 1. Early-phase clinical development predominated, with Phase I studies representing the largest proportion of completed trials (12/23, 52.2%). Four studies (17.4%) were Phase I/II trials, two (8.7%) were Phase II trials, and two (8.7%) were Phase III trials, while three studies (13.0%) did not specify a clinical phase within the registry.

**Table 1:** General characteristics of completed p53-related clinical trials (n = 23).

Characteristic	n (%)
Total studies	23 (100)
Interventional	20 (87.0)
Observational	3 (13.0)
Phase I	12 (52.2)
Phase I/II	4 (17.4)
Phase II	2 (8.7)
Phase III	2 (8.7)
Phase not reported	3 (13.0)

### 3.2. Participant enrollment

Participant enrollment information was available for 17 studies (73.9%), whereas six studies (26.1%) did not report enrollment within ClinicalTrials.gov. Availability of participant enrollment data is shown in Table 2. Across studies with available data, cumulative enrollment exceeded 2800 participants, although participant numbers varied considerably between studies, reflecting differences in study objectives and clinical development phases. Most completed studies enrolled relatively small cohorts, consistent with the predominance of early-phase dose-escalation and safety investigations.

**Table 2:** Availability of participant enrollment data.

Variable	n (%)
Studies reporting enrollment	17 (73.9)
Studies without enrollment data	6 (26.1)

### 3.3. Reporting completeness

The completeness of registry reporting varied considerably among the included studies (Table 3). Primary outcome measures were available for 15 studies (65.2%), whereas eight studies (34.8%) did not clearly report primary outcomes in the registry record. Furthermore, only five studies (21.7%) had publicly available study results posted on ClinicalTrials.gov, while the remaining 18 studies (78.3%) had no posted results despite being listed as completed.

**Table 3:** Reporting completeness of included registry records.

Registry variable	Reported n (%)	Missing/Not available n (%)
Enrolment	17 (73.9)	6 (26.1)
Primary outcome measures	15 (65.2)	8 (34.8)
Posted study results	5 (21.7)	18 (78.3)

Because of the relatively small number of studies and the heterogeneity of cancer types, interventions, and study designs, no formal comparison between studies with complete and incomplete reporting was performed.

### 3.4. Therapeutic interventions

A wide range of therapeutic strategies was identified among the completed trials. Adenoviral vector-mediated p53 gene therapy represented one of the principal therapeutic approaches identified among the included studies. Other completed trials investigated p53-directed therapeutic strategies involving immunotherapy, chemotherapy, molecularly targeted agents, biomarker-guided treatment selection, or combination treatment regimens.

Combination therapy was common, with several studies integrating p53-related interventions with chemotherapy, radiotherapy, surgery, immunotherapy, or laboratory biomarker analyses. Because individual studies frequently evaluated more than one intervention, intervention categories were not mutually exclusive and therefore exceeded the total number of included studies. Categories of therapeutic interventions evaluated across completed studies are tabulated in Table 4.

**Table 4:** Categories of therapeutic interventions evaluated across completed studies. Individual trials frequently evaluated more than one intervention; therefore, intervention categories were not mutually exclusive, and the summed number of interventions exceeds the total number of included studies (n = 23).

Intervention category	Description
Adenoviral p53 gene therapy	Ad5CMV-p53 or recombinant adenoviral p53 vectors
Chemotherapy combinations	Cytotoxic agents administered with p53-related interventions
Immunotherapy	Vaccine-based or immune-modulating strategies
Targeted therapy	Molecularly targeted therapeutic agents
Surgical procedures	Surgery combined with p53-directed treatment
Radiation therapy	Radiotherapy combined with p53-related treatment
Biomarker-guided studies	Studies evaluating p53 biomarkers or molecular profiling

### 3.5. Primary outcome measures

The primary outcome measures, which reflected the exploratory nature of the included studies, are summarized in Table 5. Safety and treatment-related toxicity were the most frequently reported endpoints, followed by determination of maximum tolerated dose, adverse-event monitoring, tumor response assessment, pharmacodynamic or biomarker evaluation, progression-free survival, feasibility, and quality-of-life outcomes. These findings indicate that completed p53-related clinical trials have primarily focused on evaluating treatment safety, biological activity, and feasibility, whereas relatively few studies assessed long-term clinical outcomes.

**Table 5:** Major categories of reported primary outcome measures.

Outcome category
Safety and toxicity
Maximum tolerated dose
Dose-limiting toxicity
Adverse events
Objective tumor response
Biomarker and pharmacodynamic evaluation
Progression-free survival
Feasibility
Quality of life

Registry reporting completeness varied considerably across the included studies. Approximately one-quarter lacked enrollment data, more than one-third lacked clearly defined primary outcome measures, and only five studies had posted results on ClinicalTrials.gov. These reporting gaps reduce transparency and limit secondary analysis, evidence synthesis, and assessment of the clinical development of p53-targeted strategies. Timely results posting and standardized reporting of enrollment and primary outcomes should therefore be prioritized in future trials.

## 4. Discussion

This registry-based analysis provides a comprehensive overview of completed clinical trials investigating p53-related therapeutic strategies in cancer registered on ClinicalTrials.gov. The findings demonstrate that clinical research in this field has been dominated by early-phase studies designed primarily to evaluate safety, tolerability, feasibility, and preliminary biological activity, rather than definitive clinical efficacy. More than half of the included studies were Phase I investigations, highlighting that the clinical development of p53-targeted interventions remains largely exploratory, despite decades of research into the biological role of TP53 in tumorigenesis.

The predominance of early-phase studies is consistent with the inherent challenges associated with translating gene- and molecular-based therapies into routine clinical practice. Restoration of p53 function has long been considered an attractive therapeutic strategy because TP53 is one of the most frequently altered tumor suppressor genes across human malignancies.^[24,26,27]^ Nevertheless, the registry data indicate that relatively few completed studies have progressed beyond early clinical development, suggesting that substantial barriers remain before these therapeutic approaches can be evaluated in large confirmatory clinical trials.

The included studies investigated a broad spectrum of malignancies, including lung, breast, ovarian, bladder, colorectal, central nervous system, and hematological cancers. This diversity reflects continued scientific interest in p53-targeted therapeutic strategies across multiple tumor types, rather than concentration within a single disease area.^[4,28]^

Another finding of the present analysis was the considerable variability in registry reporting completeness. Although participant enrollment was available for most studies, approximately one-quarter of the included trials did not report enrollment data, and more than one-third lacked clearly defined primary outcome measures within the registry. Furthermore, most completed studies had not posted study results on ClinicalTrials.gov. Limited reporting of trial outcomes reduces transparency and restricts opportunities for secondary analyses, evidence synthesis, and evaluation of the overall progress of p53-targeted clinical research. Improving the completeness and timeliness of registry reporting should therefore be considered an important priority for future studies.

The intervention strategies identified across the included trials illustrate the evolution of p53-directed research over time. Early investigations primarily evaluated adenoviral vector-mediated p53 gene delivery either as monotherapy or in combination with chemotherapy, surgery, or radiotherapy.^[29,30]^ More recent studies expanded to include molecularly targeted therapies, immunotherapeutic approaches, and biomarker-guided treatment strategies involving TP53 alterations.^[31–33]^ Some registered protocols combined these approaches with other anticancer interventions or incorporated biomarkers into treatment selection. However, registry information describes planned study designs and does not establish whether the registered interventions were implemented as intended or resulted in improved clinical outcomes.

Another important observation is the emphasis placed on safety-related endpoints. Most reported primary outcome measures focused on adverse events, dose-limiting toxicity, maximum tolerated dose, and treatment feasibility, reflecting the predominance of early-phase clinical development. Only a limited number of studies reported efficacy-related endpoints, and the registry records alone do not provide sufficient information to assess treatment effectiveness, survival benefit, or comparative clinical performance. Consequently, the findings of the present study should be interpreted as a description of clinical trial characteristics rather than evidence of therapeutic efficacy.

The present study has several strengths. It provides an updated overview of completed ClinicalTrials.gov studies investigating p53-related therapeutic strategies and summarizes important characteristics, including study phase, intervention types, enrollment, reporting completeness, and primary outcome measures. By highlighting current research patterns and reporting practices, the analysis offers a useful reference for investigators planning future clinical studies in this field.

However, several limitations should be considered when interpreting these findings. First, the analysis was limited to studies registered on ClinicalTrials.gov and may not include completed trials registered in other international databases. Second, registry records varied considerably in reporting completeness, with missing information for enrollment, primary outcomes, and posted study results in several studies. Third, the heterogeneity of cancer types, intervention strategies, and study designs precluded quantitative comparisons between studies. A formal risk-of-bias assessment was not conducted because the unit of analysis was the registry record rather than a published clinical study report. Although reporting completeness was evaluated according to the availability of enrollment data, primary outcome measures, and posted study results, this assessment should not be interpreted as a measure of trial methodological quality or risk of bias. Finally, because this investigation was designed as a descriptive registry analysis, no inferential statistical analyses or pooled evaluations of clinical efficacy were performed. Accordingly, the findings should be interpreted as an overview of completed p53-related clinical trials rather than an assessment of treatment effectiveness.

Overall, the present analysis demonstrates that completed p53-related clinical trials remain concentrated in early clinical development and continue to emphasize safety, feasibility, and preliminary biological investigation. Future research would benefit from improved reporting transparency, greater availability of trial results within public registries, and progression toward larger, well-designed late-phase studies capable of evaluating the clinical effectiveness of p53-targeted therapeutic strategies.

## 5. Conclusion

This registry-based analysis demonstrates that completed clinical trials investigating p53-targeted therapeutic strategies remain predominantly concentrated in early clinical development. Most studies focused on safety, feasibility, and preliminary biological activity, while relatively few progressed to late-phase clinical evaluation. Incomplete reporting of enrollment, primary outcome measures, and study results continues to limit comprehensive assessment of the current evidence base. Future research should prioritize timely and mandatory results posting, standardized reporting of primary outcomes and enrollment data, and adequately powered late-phase trials to better define the clinical role of p53-targeted therapeutic strategies across different cancer types.

## 6. Limitations

This analysis was limited to completed studies registered on ClinicalTrials.gov and may not capture unregistered or unpublished trials. Many studies lacked detailed result postings, restricting quantitative efficacy comparisons. Additionally, heterogeneity in cancer types and interventions prevented pooled analysis.

## Acknowledgments

The authors extend their appreciation to Umm Al-Qura University, Saudi Arabia for funding this research work through grant number: 26UQU4420038GSSR09.

## Author contributions

**Conceptualization:** Nasser M. Alorfi.

**Data curation:** Nasser M. Alorfi.

**Formal analysis:** Nasser M. Alorfi.

**Funding acquisition:** Nasser M. Alorfi.

**Investigation:** Nasser M. Alorfi.

**Methodology:** Nasser M. Alorfi.

**Project administration:** Nasser M. Alorfi.

**Resources:** Nasser M. Alorfi.

**Software:** Nasser M. Alorfi.

**Supervision:** Nasser M. Alorfi.

**Validation:** Nasser M. Alorfi.

**Visualization:** Nasser M. Alorfi.

**Writing – review & editing:** Nouf M. Alourfi.


