# Supplementary material for: Completed clinical trials of p53-targeted therapeutic strategies in cancer: A cross-sectional ClinicalTrials.gov registry analysis
Source: Medicine (Baltimore). 2026 Oct 16;105(42):e50949. doi: 10.1097/MD.0000000000050949 (PMC13680912; doi:10.1097/MD.0000000000050949)
Supplement: Supplementary file 1 [file medi-105-e50949-s001.docx]

Supplementary File S1 (updated from clinicaltrials.gov on 1^st^ July 2025 )

| **NCT Number** | **Study Title** | **Brief Summary** | **Study Results** | **Conditions** | **Interventions** | **Primary Outcome Measures** | **Sex** | **Phases** | **Enrollment** | **Study Type** |
| --- | --- | --- | --- | --- | --- | --- | --- | --- | --- | --- |
| **NCT00003167** | Gene Therapy in Treating Patients With Advanced Bladder Cancer | Phase I trial to study the effectiveness of gene therapy in treating patients with advanced bladder cancer. Inserting the p53 gene into a person's bladder cancer cells may improve the body's ability to fight cancer | NO | Recurrent Bladder Cancer\|Stage I Bladder Cancer\|Stage II Bladder Cancer\|Stage III Bladder Cancer\|Stage IV Bladder Cancer\|Transitional Cell Carcinoma of the Bladder | BIOLOGICAL: Ad5CMV-p53 gene | Maximum-tolerated dose as assessed by NCI Common Terminology Criteria (CTC) version 2.0, 4 weeks\|Safety and toxicity of adenovirus p53 (Ad-p53) gene therapy according to NCI CTC version 2.0, Up to 1 year after completion of treatment | ALL | PHASE1 | 24 | INTERVENTIONAL |
| **NCT00003450** | Gene Therapy in Treating Patients With Advanced Recurrent or Persistent Ovarian Cancer or Primary Peritoneal Cancer | RATIONALE: Inserting the p53 gene into a person's cancer cells may improve the body's ability to fight cancer or make the cancer more sensitive to chemotherapy.  PURPOSE: Phase I trial to study the effectiveness of gene therapy using the p53 gene in treating patients with advanced recurrent or persistent ovarian cancer or primary peritoneal cavity cancer. | NO | Ovarian Cancer\|Peritoneal Cavity Cancer | BIOLOGICAL: Ad5CMV-p53 gene |  | FEMALE | PHASE1 |  | INTERVENTIONAL |
| **NCT00003649** | Gene Therapy in Treating Patients With Non-small Cell Lung Cancer That Cannot Be Surgically Removed | RATIONALE: Exposing tumor cells to the p53 gene may improve the body's ability to fight non-small cell lung cancer.  PURPOSE: Phase I trial to study the effectiveness of gene therapy in treating patients who have non-small cell lung cancer that cannot be surgically removed. | NO | Lung Cancer | BIOLOGICAL: Ad5CMV-p53 gene |  | ALL | PHASE1 |  | INTERVENTIONAL |
| **NCT00004038** | Gene Therapy Plus Chemotherapy in Treating Patients With Breast Cancer | Phase I trial to study the effectiveness of gene therapy plus chemotherapy in treating patients who have breast cancer. Inserting the p53 gene into a person's cancer cells may improve the body's ability to fight cancer or make the cancer more sensitive to chemotherapy. Combining chemotherapy with gene therapy may kill more tumor cells. | NO | Breast Cancer | BIOLOGICAL: Ad5CMV-p53 gene\|DRUG: chemotherapy |  | ALL | PHASE1 | 20 | INTERVENTIONAL |
| **NCT00004041** | Gene Therapy in Treating Patients With Recurrent Malignant Gliomas | RATIONALE: Inserting the gene for adenovirus p53 into a person's tumor may improve the body's ability to fight cancer.  PURPOSE: Phase I trial to study the effectiveness of gene therapy in treating patients who have recurrent malignant gliomas. | NO | Brain and Central Nervous System Tumors | BIOLOGICAL: Ad5CMV-p53 gene\|PROCEDURE: conventional surgery |  | ALL | PHASE1 |  | INTERVENTIONAL |
| **NCT00004225** | Gene Therapy Plus Radiation Therapy in Treating Patients With Non-Small Cell Lung Cancer | RATIONALE: Inserting the gene for p53 into a person's cancer cells may improve the body's ability to fight cancer. Radiation therapy uses high-energy x-rays to damage tumor cells.  PURPOSE: Phase I trial to study the effectiveness of gene therapy plus radiation therapy in treating patients who have non-small cell lung cancer. | NO | Lung Cancer | BIOLOGICAL: Ad5CMV-p53 gene\|RADIATION: radiation therapy |  | ALL | PHASE1 |  | INTERVENTIONAL |
| **NCT00003588** | Gene Therapy in Treating Patients With Ovarian Cancer | Phase I trial to study the effectiveness of gene therapy in treating patients with ovarian cancer that has not responded to previous treatment. Inserting the p53 gene into a person's cancer cells may improve the body's ability to fight cancer or make the cancer cells more sensitive to treatment. | NO | Ovarian Cancer | BIOLOGICAL: Ad5CMV-p53 gene\|PROCEDURE: laparoscopic surgery |  | FEMALE | PHASE1 | 30 | INTERVENTIONAL |
| **NCT00064103** | Gene Therapy in Preventing Cancer in Patients With Premalignant Carcinoma of the Oral Cavity or Pharynx | This phase I/II trial is studying the side effects and best dose of gene therapy and to see how well it works in preventing cancer in patients with premalignant carcinoma of the oral cavity or pharynx. Inserting the p53 gene into a person's tumor cells may improve the body's ability to kill the tumor cells | NO | Lip and Oral Cavity Cancer\|Oropharyngeal Cancer\|Stage 0 Lip and Oral Cavity Cancer\|Stage 0 Oropharyngeal Cancer\|Tongue Cancer | BIOLOGICAL: Ad5CMV-p53 gene\|OTHER: laboratory biomarker analysis | Frequency of adverse events, Up to 15 years\|Severity of adverse events graded using the CTCAE version 3.0, Up to 15 years\|Maximum tolerated dose of Ad5CMV-p53 gene, Evaluated by the frequency and relationship of dose-limiting toxicities, if any, experienced by patients during dose escalation., 6 months\|Pharmacodynamics evaluated by examining the injected precancerous lesion for induction of apoptosis and expression of the p53 protein, Presented using descriptive statistics, frequency tabulations, and graphical displays over time by treatment cohort., 168 days | ALL | PHASE1\|PHASE2 | 51 | INTERVENTIONAL |
| **NCT00004080** | Gene Therapy in Treating Patients With Recurrent or Progressive Brain Tumors | RATIONALE: Inserting the gene for p53 into a person's brain cells may improve the body's ability to fight cancer.  PURPOSE: Phase I trial to study the effectiveness of p53 gene therapy with SCH-58500 in treating patients who have recurrent, or progressive glioblastoma multiforme, anaplastic astrocytoma, or anaplastic mixed glioma that can be removed during surgery. | NO | Brain and Central Nervous System Tumors | BIOLOGICAL: recombinant adenovirus-p53 SCH-58500\|PROCEDURE: conventional surgery |  | ALL | PHASE1 |  | INTERVENTIONAL |
| **NCT00044993** | Chemotherapy Combined With Gene Therapy in Treating Patients Who Have Stage III or Stage IV Breast Cancer | RATIONALE: Drugs used in chemotherapy use different ways to stop tumor cells from dividing so they stop growing or die. Inserting the p53 gene into the tumor may increase the effectiveness of a chemotherapy drug by making tumor cells more sensitive to the drug. Combining chemotherapy with gene therapy may kill more tumor cells.  PURPOSE: Phase II trial to study the effectiveness of combining chemotherapy with gene therapy in treating patients who have stage III or stage IV breast cancer. | NO | Breast Cancer | BIOLOGICAL: Ad5CMV-p53 gene\|DRUG: docetaxel\|DRUG: doxorubicin hydrochloride\|PROCEDURE: conventional surgery\|PROCEDURE: neoadjuvant therapy |  | ALL | PHASE2 |  | INTERVENTIONAL |
| **NCT00897663** | Improving the Selection of Patients With Glioblastoma Multiforme for Treatment With Epidermal Growth Factor Receptor Inhibitor Therapies | RATIONALE: Studying samples of tissue from patients with cancer in the laboratory may help doctors identify and learn more about biomarkers related to cancer. It may also help doctors predict how patients will respond to treatment.  PURPOSE: This laboratory study is looking at tissue samples from patients with glioblastoma multiforme to identify biomarkers that may improve the selection of patients for epidermal growth factor receptor inhibitor therapies. | NO | Brain and Central Nervous System Tumors | GENETIC: gene expression analysis\|GENETIC: microarray analysis\|GENETIC: protein expression analysis\|OTHER: diagnostic laboratory biomarker analysis\|OTHER: immunohistochemistry staining method | Molecular characteristics that predict for overall survival and progression-free survival, Up to 24 months | ALL |  | 56 | OBSERVATIONAL |
| **NCT00049218** | Chemotherapy Followed By Vaccine Therapy in Treating Patients With Extensive-Stage Small Cell Lung Cancer | RATIONALE: Drugs used in chemotherapy use different ways to stop tumor cells from dividing so they stop growing or die. Vaccines made from a gene-modified virus may make the body build an immune response to kill tumor cells. Combining vaccine therapy with chemotherapy may kill more tumor cells.  PURPOSE: Phase I/II trial to study the effectiveness of chemotherapy followed by adenovirus p53 vaccine therapy in treating patients who have extensive-stage small cell lung cancer. | NO | Lung Cancer | BIOLOGICAL: Autologous dendritic cell-adenovirus p53 vaccine\|DRUG: Carboplatin\|DRUG: Etoposide | Rate of Toxicity of the Ad-p53 DC Vaccine, To evaluate the toxicity of the Ad-p53 dendritic cell (DC) vaccine. While there is no expected toxicity from the Ad-p53 vaccine, there may be unforeseen adverse effects. Patients will be monitored for toxicity, particularly for evidence of autoimmunity. Complete blood counts (CBCs) to monitor for hematologic toxicity, serum creatinine to monitor for renal toxicity, liver function tests (LFTs) to monitor for hepatic toxicity, and a standard clinical toxicity will be performed every other week throughout the period of immunization. In addition, a medical history and physical examination will be performed on a monthly basis., 4 years | ALL | PHASE1\|PHASE2 | 56 | INTERVENTIONAL |
| **NCT00280761** | Biomarkers in Patients With Rectal Cancer Undergoing Chemotherapy and Radiation Therapy | RATIONALE: Studying samples of tumor tissue from patients with cancer in the laboratory may help doctors learn more about changes that occur in DNA and identify biomarkers related to cancer. It may also help doctors understand how patients respond to treatment.  PURPOSE: This clinical trial is studying biomarkers in patients with rectal cancer undergoing chemotherapy and radiation therapy. | NO | Colorectal Cancer | DRUG: capecitabine\|DRUG: 5-fluorouracil\|PROCEDURE: Surgical Resection\|RADIATION: Radiation therapy | Activation of NF-kappa B in response to treatment with external beam radiotherapy, 6-8 weeks after chemoradiation\|Correlation of NF-kappa B pathway activation with therapeutic outcomes, 6-8 weeks after chemoradiation | ALL |  | 47 | OBSERVATIONAL |
| **NCT00017095** | Biomarker (p53 Gene) Analysis and Combination Chemotherapy Followed by Radiation Therapy and Surgery in Treating Women With Large Operable or Locally Advanced or Inflammatory Breast Cancer | RATIONALE: Drugs used in chemotherapy work in different ways to stop tumor cells from dividing so they stop growing or die. Currently patients with breast cancer are treated with one of several very similar combinations of drugs. Analysis of biomarkers in tumor tissue may help doctors predict how well patients with breast cancer will respond to treatment and help doctors choose the best drug regimen to treat each patient.  PURPOSE: This randomized phase III trial is studying giving different regimens of chemotherapy and comparing how well they work in treating women with large operable or locally advanced or inflammatory breast cancer. This study is also looking at whether analyzing a specific biomarker (p53) in tumor tissue may help doctors predict how well patients will respond to treatment and help doctors choose the best drug to treat each patient. | NO | Breast Cancer | BIOLOGICAL: filgrastim\|DRUG: cyclophosphamide\|DRUG: docetaxel\|DRUG: epirubicin hydrochloride\|DRUG: fluorouracil\|GENETIC: microarray analysis\|OTHER: immunohistochemistry staining method\|OTHER: laboratory biomarker analysis\|PROCEDURE: biopsy\|PROCEDURE: conventional surgery\|PROCEDURE: neoadjuvant therapy\|RADIATION: radiation therapy | Progression-free survival, from randomization till first evidence of progression | FEMALE | PHASE3 | 1856 | INTERVENTIONAL |
| **NCT00496860** | Safety and Efficacy Study of ALT-801 to Treat Progressive Metastatic Malignancies | This is a Phase 1, open-labeled, non-randomized, multi-center, competitive enrollment and dose-escalation study of ALT-801, the study drug. The purpose of this study is to evaluate the safety, determine the maximum-tolerated dose (MTD) and characterize the pharmacokinetic profile of ALT-801 in previously treated patients with progressive metastatic malignancies. ALT-801, a recombinant fusion protein with a interleukin-2 (IL-2) component, has a targeting mechanism that recognizes tumor cells with a specific tumor marker. | YES | Progressive Metastatic Malignancies | BIOLOGICAL: ALT-801 | The Safety and Toxicity of ALT-801 in Patients With Progressive Metastatic Malignancies, Number of serious adverse events per cohort, 18 months\|The Maximum-tolerated Dose (MTD) of ALT-801, Number of dose limiting toxicities (DLTs). A DLT is a toxicity that results in patient withdrawal from the study as defined in the protocol., 18 months | ALL | PHASE1 | 26 | INTERVENTIONAL |
| **NCT01326871** | A Study of ALT-801 in Combination With Cisplatin and Gemcitabine in Muscle Invasive or Metastatic Urothelial Cancer | This is a Phase Ib/II, open-label, multi-center, competitive enrollment and dose-escalation study of ALT-801 in a biochemotherapy regimen either containing cisplatin and gemcitabine or containing gemcitabine alone in patients who have muscle invasive or metastatic urothelial cancer of bladder, renal pelvis, ureters and urethra. The purpose of this study is to evaluate the safety, determine the maximum tolerated dose (MTD) and the recommended dose (RD), and assess the anti-tumor response of ALT-801 in combination with cisplatin and gemcitabine or ALT-801 in combination with gemcitabine alone. The pharmacokinetic profile of ALT-801 in combination with cisplatin and gemcitabine will also be assessed. The study includes a dose escalation phase (Phase Ib) and a dose expansion phase (Phase II). Phase II has two treatment groups, Expansion Group 1 and Expansion Group 2. Expansion Group 2 is for platinum-refractory patients, consisting of two treatment arms based on the patient's renal function. Patients will enroll to Expansion Group 2 after stage 1 of the Group 1 expansion is complete. | YES | Transitional Cell Carcinoma of Bladder\|Urethra Cancer\|Ureter Cancer\|Malignant Tumor of Renal Pelvis | DRUG: Cisplatin\|DRUG: Gemcitabine\|BIOLOGICAL: ALT-801 | Maximum Tolerated Dose (MTD) and/or the Recommended Dose (RD) for Dose Expansion of ALT-801 in Combination With Cisplatin and Gemcitabine or ALT-801 in Combination With Gemcitabine Alone, 8 weeks\|Number of Participants With Adverse Events, Number of AEs that occur or worsen after the first dose of study treatment, 8 weeks\|Objective Response Rate in Treated Patients, Objective response rate (ORR) is defined as confirmed complete response (CR) or partial response (PR) using Response Evaluation Criteria in Solid Tumors \[RECIST V1.0\]: a complete response is the disappearance of all target lesions; a partial response is defined as at least a 30% decrease in the sum of diameters of target lesions, taking as reference the baseline sum diameters. ORR = CR + PR., 12 weeks | ALL | PHASE1\|PHASE2 | 68 | INTERVENTIONAL |
| **NCT00898755** | Collecting and Storing Tissue From Young Patients With Cancer | This laboratory study is collecting and storing tissue, blood, and bone marrow samples from young patients with cancer. Collecting and storing samples of tissue, blood, and bone marrow from patients with cancer to study in the laboratory may help doctors learn more about changes that may occur in DNA and identify biomarkers related to cancer. | NO | Acute Lymphoblastic Leukemia\|Acute Myeloid Leukemia\|Central Nervous System Neoplasm\|Ewing Sarcoma\|Germ Cell Tumor\|Leukemia\|Lymphoma\|Malignant Neoplasm\|Neuroblastoma\|Osteosarcoma\|Retinoblastoma\|Rhabdoid Tumor\|Rhabdomyosarcoma\|Soft Tissue Sarcoma | OTHER: Cytology Specimen Collection Procedure\|OTHER: Laboratory Biomarker Analysis | Establishment and banking of cell lines and/or xenografts from pediatric patients with cancer, Up to 14 years\|Establishment of continuous cell lines, under carefully controlled conditions, from pediatric patients with cancer, Up to 14 years\|Establishment of transplantable xenografts in immunocompromised mice from tumor cells that are difficult to establish as continuous cell lines in vitro, Up to 14 years\|Creation of a bank of cell lines and generation of sufficient vials of cryopreserved cells for distribution to investigators with approved COG biology protocols, Up to 14 years\|Characterization of cell lines from childhood cancers with respect to DNA PCR molecular HLA profile as a "fingerprint" of original cell line identity, Up to 14 years\|Characterization of cell lines for the ability for sustained growth in tissue culture and/or as mouse xenografts, Up to 14 years\|Characterization of cell lines for mycoplasma contamination, Up to 14 years\|Characterization of cell lines for expression of molecular makers that confirm the tumor-type of the cell line and the immortal nature of the cells (telomerase) and the expression of molecular markers that may correlate with drug resistance, Up to 14 years | ALL |  | 213 | OBSERVATIONAL |
| **NCT04277442** | Testing Nivolumab in Combination With Decitabine and Venetoclax in Patients With Newly Diagnosed TP53 Gene Mutated Acute Myeloid Leukemia | This trial studies the side effects of nivolumab in combination with decitabine and venetoclax and to see how well they work in treating patients with TP53-mutated acute myeloid leukemia. Immunotherapy with monoclonal antibodies, such as nivolumab, may help the body's immune system attack the cancer, and may interfere with the ability of tumor cells to grow and spread. Drugs used in chemotherapy, such as decitabine and venetoclax, work in different ways to stop the growth of cancer cells, either by killing the cells, by stopping them from dividing, or by stopping them from spreading. This study is being done to find out whether giving nivolumab, decitabine, and venetoclax is better or worse than the usual approach for TP53-mutated acute myeloid leukemia. | YES | Acute Myeloid Leukemia | DRUG: Decitabine\|BIOLOGICAL: Nivolumab\|DRUG: Venetoclax | Incidence of Adverse Events, Toxicity will be graded and reported according to the revised National Cancer Institute Common Terminology Criteria for Adverse Events version 5.0., Up to day 15 of cycle 1 (each cycle is 28 days)\|Number of Patients That Are Able to Complete 3 Cycles of Therapy, Feasibility will be met if 10 of 13 patients are able to complete 3 cycles of therapy., Up to day 15 of cycle 1 (each cycle is 28 days)\|Response, Response will be defined as being able to achieve complete remission, complete remission with incomplete count recovery, or complete remission with incomplete hematological recovery by cycle 3., Up to day 15 of cycle 1 (each cycle is 28 days) | ALL | PHASE1 | 1 | INTERVENTIONAL |
| **NCT01029873** | QUILT-2.008: Study of ALT-801 With Cisplatin in Patients With Metastatic Melanoma | This is a Phase Ib/II, open-label, multi-center, competitive enrollment and dose-escalation study of ALT-801 combined with cisplatin. The purpose of this study is to evaluate the safety, determine the Maximum-Tolerated Dose (MTD), and characterize the pharmacokinetic profile of ALT-801 given with cisplatin in patients who are chemotherapy naïve and have metastatic melanoma that is considered surgically incurable. The anti-tumor responses of ALT-801 with cisplatin will also be assessed in this trial. | NO | Metastatic Melanoma | DRUG: Cisplatin\|BIOLOGICAL: ALT-801 | To evaluate the safety of the ALT-801-Cisplatin regimen., 12 months\|To assess the objective response (OR) which includes CR and PR., 3 months\|To assess the clinical benefit (CB) of the ALT-801-Cisplatin regimen which includes CR, PR and SD., 3 months\|To determine the MTD of the ALT-801-Cisplatin regimen., 7 weeks | ALL | PHASE1\|PHASE2 | 25 | INTERVENTIONAL |
| **NCT02090114** | RE-sensitizing With Supraphysiologic Testosterone to Overcome REsistance (The RESTORE Study) | Single-arm, single site, open label study of the effects of parenteral testosterone followed by enzalutamide, abiraterone or castration-only therapy in men with metastatic CRPC who previously progressed on one of these forms of therapy. The study will enroll four cohorts of patients: men with metastatic CRPC who have progressed on enzalutamide (Cohort A; n=30); men with metastatic CRPC who have progressed on abiraterone acetate (Cohort B; n=30); men with metastatic CRPC who have progressed on first line castration-only therapy (Cohort C; n=30); men with metastatic CRPC with inactivating somatic or germline mutations in ≥2 of the genes TP53, PTEN, or RB1 (Cohort D; n=20). | YES | Prostate Cancer | DRUG: Testosterone cypionate\|DRUG: Testosterone Enanthate\|DRUG: Abiraterone acetate\|DRUG: Enzalutamide | Prostate Specific Antigen (PSA) Response to Bipolar Androgen Therapy (BAT), Number of participants with ≥50% PSA reduction from pre-BAT baseline level, up to 18 months\|PSA Response to Enzalutamide or Abiraterone Acetate Post Bipolar Androgen Therapy, Number of participants with ≥50% PSA reduction after enzalutamide or abiraterone acetate post-BAT from baseline, up to 24 months | MALE | PHASE2 | 112 | INTERVENTIONAL |
| **NCT00423865** | Cisplatin and Everolimus in Treating Patients With Advanced Solid Tumors | RATIONALE: Drugs used in chemotherapy, such as cisplatin, work in different ways to stop the growth of tumor cells, either by killing the cells or by stopping them from dividing. Everolimus may stop the growth of tumor cells by blocking some of the enzymes needed for cell growth. Everolimus may also help cisplatin work better by making tumor cells more sensitive to the drug. Giving cisplatin together with everolimus may kill more tumor cells.  PURPOSE: This phase I trial is studying the side effects and best dose of everolimus when given together with cisplatin in treating patients with advanced solid tumors or recurrent or metastatic solid tumors. | NO | Unspecified Adult Solid Tumor, Protocol Specific | DRUG: cisplatin\|DRUG: everolimus\|GENETIC: gene expression analysis\|OTHER: immunohistochemistry staining method\|OTHER: laboratory biomarker analysis\|OTHER: pharmacological study\|PROCEDURE: biopsy | Recommended phase II dose of everolimus, 1 year | ALL | PHASE1 | 30 | INTERVENTIONAL |
| **NCT02980731** | A Study Evaluating the Impact of Venetoclax on the Quality of Life for Subjects With Relapsed (Your Cancer Has Come Back) or Refractory (no Response to Previous Cancer Treatments) Chronic Lymphocytic Leukemia (CLL) While Receiving Venetoclax Monotherapy (a Single Agent). | The purpose of this open-label, single-arm study was to evaluate the impact of venetoclax on the quality of life of participants including those with with relapsed/refractory (R/R) chronic lymphocytic leukemia (CLL; a type of cancer affecting the blood and the bone marrow) with or without the 17p deletion or TP53 mutation, including participants with an unknown status, as well as R/R CLL participants who had been previously treated with B-cell receptor inhibitor (BCRi) therapy. The starting dose of venetoclax was 20 mg once daily. The dose must have been gradually increased over a period of 5 weeks up to the daily dose of 400 mg. Participants may have continued receiving venetoclax for up to 2 years. After the treatment period, participants may have continued on into a 2-year follow-up period. | YES | Chronic Lymphocytic Leukemia (CLL) | DRUG: Venetoclax | Mean Change From Baseline to Week 48 in Global Health Status/Quality of Life (GHS/QoL) Subscale of the European Organization for Research and Treatment of Cancer Quality of Life Core Questionnaire (EORTC QLQ-C30), EORTC QLQ-C30 is a 30-item participant self-report questionnaire composed of both multi-item and single scales, including a global health status/quality of life (GHS/QoL) scale. Participants rate items and a score ranging from 0 to 100 is calculated. A higher score on the global health status/quality of life scale indicates a better level of functioning, and positive changes from baseline indicate improvement. A change of 5 - 10 points is considered a small change, and a change of 10 - 20 points is considered a moderate change., Baseline, Week 48 | ALL | PHASE3 | 210 | INTERVENTIONAL |
| **NCT01212380** | Study of Carfilzomib in Chronic Lymphocytic Leukemia (CLL), Small Lymphocytic Lymphoma (SLL) or Prolymphocytic Leukemia (PLL) | RATIONALE:  Carfilzomib may stop the growth of cancer cells by blocking some of the enzymes needed for cell growth.  PURPOSE:  This phase I trial is studying the side effects and the best dose of carfilzomib in treating patients with relapsed or refractory chronic lymphocytic leukemia(CLL),small lymphocytic lymphoma(SLL), or prolymphocytic leukemia (PLL). | NO | B-cell Chronic Lymphocytic Leukemia\|Hematopoietic/Lymphoid Cancer\|Prolymphocytic Leukemia\|Recurrent Small Lymphocytic Lymphoma\|Refractory Chronic Lymphocytic Leukemia | DRUG: carfilzomib\|OTHER: Cytokine Assessment\|OTHER: Pharmacodynamic Studies\|OTHER: Proteosome Inhibition Assessment\|OTHER: Pharmacogenomic Studies | Determine safety of carfilzomib by evaluating the toxicity profile., The safe use of carfilzomib will be assessed by:  * Determining the dose limiting toxicity and maximal tolerated dose of carfilzomib in patients with relapsed or refractory chronic lymphocytic leukemia (CLL)/small lymphocytic leukemia (SLL) and prolymphocytic leukemia (PLL)  * To evaluating the toxicity profile of carfilzomib in relapsed or refractory chronic lymphocytic leukemia (CLL)/small lymphocytic leukemia (SLL) and prolymphocytic leukemia (PLL), Up to 24 months | ALL | PHASE1 | 21 | INTERVENTIONAL |
